# Sarcopenic Obesity in Facioscapulohumeral Muscular Dystrophy

**DOI:** 10.3389/fphys.2020.01008

**Published:** 2020-08-12

**Authors:** Kathryn Vera, Mary McConville, Michael Kyba, Manda Keller-Ross

**Affiliations:** ^1^Division of Rehabilitation Science, University of Minnesota, Minneapolis, MN, United States; ^2^Health and Human Performance Department, University of Wisconsin—River Falls, River Falls, WI, United States; ^3^College of Saint Benedict, St. Joseph, MN, United States; ^4^Lillehei Heart Institute and Department of Pediatrics, University of Minnesota, Minneapolis, MN, United States; ^5^Division of Physical Therapy, University of Minnesota, Minneapolis, MN, United States

**Keywords:** FSHD, muscular dystrophy, sarcopenic obesity, appendicular lean mass index, body composition

## Abstract

**Background:**

Sarcopenic obesity has been observed in people with neuromuscular impairment, and is linked to adverse health outcomes. It is unclear, however, if sarcopenic obesity develops in adults with facioscapulohumeral muscular dystrophy (FSHD).

**Methods:**

The purpose of this study was to determine if adults with FSHD meet criteria for sarcopenic obesity (appendicular lean mass index (ALMI) scores of < 7.26 or 5.45 kg/m^2^; % fat mass (FM) ≥ 28 or 40% in men/women). Ten people with FSHD (50 ± 11 years, 2 females) and ten age/sex-matched controls (47 ± 13 years, 2 females) completed one visit, which included a full-body dual-energy x-ray absorptiometry (DXA) scan. Regional and whole body total mass, fat mass (FM), and lean mass (LM) were collected and body mass index (BMI) and sarcopenia measures were computed.

**Results:**

People with FSHD and controls had a similar whole body total mass (84.5 ± 12.9 vs. 81.8 ± 13.5 kg, respectively, *p* = 0.65). Though BMI was 2% lower in the FSHD group (*p* = 0.77), the % FM was 46% higher in FSHD, compared with controls (*p* < 0.01). In addition, ALM volume was 23% lower (*p* = 0.02) and ALMI was 27% lower in FSHD compared with controls (*p* < 0.01). Whole body LM trended to be lower in FSHD vs. controls (*p* = 0.05), and arm and leg LM were both lower in FSHD compared with controls (*p* < 0.05). Furthermore, the % LM was 18% lower in FSHD vs. controls (*p* < 0.01). FSHD participants exhibited greater total body FM (*p* < 0.01) and total leg FM (*p* < 0.01), but were similar in volume of total arm FM compared with controls (*p* = 0.09).

**Conclusion:**

Findings from this study suggest that people with FSHD, although similar in BMI and total body mass compared with controls, commonly meet the definition of sarcopenic obesity. Adults with co-existing FSHD and sarcopenic obesity may be at risk for significant impairments in quality of life, and encounter additional challenges in the management of FSHD manifestations.

## Introduction

Facioscapulohumeral muscular dystrophy (FSHD) is one of the most common dominantly-inherited muscular dystrophies, with prevalence frequencies ranging from 1:15,000 to 1:21,000 worldwide (Deenen et al., 2014). Classically, FSHD manifests in progressive, often asymmetrical muscular weakness, most prominently in the face, shoulder girdle, and upper-arm region (Statland and Tawil, 2016; Wang and Tawil, 2016). Unlike in other forms of muscular dystrophy, in which the presence of cardiomyopathy and pulmonary impairment frequently results in a heightened mortality rate (McNally et al., 2015), life expectancy among individuals with FSHD appears to be preserved (Statland and Tawil, 2016). However, impairments in functional capacity, as measured by a decreased capacity for independent ambulation (Wang and Tawil, 2016), and a greater reliance on assistive devices among older adults with FSHD (>50 years), have been reported in this population (Statland and Tawil, 2014). It is speculated that this functional impairment may be partially driven by alterations in body composition, which likewise have been linked to high rates of skeletal muscle atrophy (Statland and Tawil, 2016; Wang and Tawil, 2016) and corresponding increases in fatty tissue infiltration of the muscular compartments (Janssen et al., 2014). For example, Janssen et al. (2014) demonstrated that up to 26% of individuals with FSHD may experience severe rates of fatty infiltration, whereby as much as 75% of lean tissue in certain muscular compartments is replaced by fat mass, an observation that may have clinical and functional implications for people with FSHD.

With the manifestation of muscular atrophy and increased proportion of fat mass, it is likely that people with FSHD exhibit a medical condition known as sarcopenic obesity. Sarcopenic obesity combines the key features of sarcopenia [losses in muscle mass, declining strength, and/or impairments in physical performance (Cruz-Jentoft et al., 2010)] with an increased presence of adiposity (Prado et al., 2012). Adults who meet diagnostic criteria for sarcopenic obesity have been reported to exhibit an increased risk of mortality (hazard ratio: 1.44) as compared to control counterparts (Androga et al., 2017), and show a greater propensity toward physical disability (Baumgartner, 2000). It is believed that sarcopenic obesity contributes to physical disability via a combination of concurrent changes, including the loss and atrophy of type II muscle fibers (Walrand et al., 2011) and a greater propensity toward fatty infiltration of skeletal muscle (Goodpaster et al., 2006). These skeletal muscle alterations lead to impairments in the contractile strength of type I and II muscle fibers (Hamrick et al., 2016), and overall reductions in muscular power.

While sarcopenic obesity has been noted among individuals with various types of muscular dystrophy [i.e., Duchenne, Becker, and Ullrich congenital muscular dystrophies (Merlini et al., 2014)], it is unclear if people with FSHD exhibit this condition. By identifying sarcopenic obesity as a potential comorbidity of FSHD, the development of effective preventative and therapeutic strategies designed to address the condition may be incorporated as part of the medical treatment plan, thereby leading to gains in functional capacity, a greater ability to perform activities of daily living (ADLs), and an overall improvement in quality of life. Therefore, we aimed to determine if people with FSHD meet the diagnostic criteria for sarcopenic obesity. We hypothesized that the anatomic characteristics associated with the presence of sarcopenic obesity are more pronounced among people with FSHD, as compared to age- and sex-matched controls.

## Materials and Methods

### Subjects

Ten adults with genetically-confirmed FSHD and ten age- and sex-matched healthy control participants (*n* = 20 combined; men: 16, women: 4) completed the study. Inclusion criteria consisted of an age of ≥ 18 years, and no prior history of cardiovascular, pulmonary, orthopedic, or neuromuscular disorders other than FSHD; female participants were excluded if they were currently pregnant or breastfeeding (Dewey, 1997; Lof et al., 2005). Physical activity levels were assessed via the Modified Minnesota Leisure Time Physical Activity Questionnaire, and reported as an activity metabolic index score (Richardson et al., 1994). Severity of disease burden among individuals with FSHD was evaluated through completion of the FSHD Health Index survey, whereby a score of 100 reflects the highest disease, and 0 reflects no disease burden (Johnson et al., 2012; Hamel et al., 2019). The study was approved by the University of Minnesota Institutional Review Board, and conducted in accordance with the Declaration of Helsinki.

### Experimental Protocol

Study participants attended one study session, which included a written informed consent following a description of the study design and a total-body dual-energy x-ray absorptiometry (DXA) scan (Lunar iDXA, GE Healthcare, Chicago, IL, United States); female participants took a urine human chorionic gonadotropin (hCG) test (Clinical Guard, Atlanta, GA, United States) to confirm the absence of pregnancy.

### Data Collection Techniques

Body composition was obtained from the DXA scan; an estimation of regional and whole body total mass [kilograms (kg)], fat mass [FM (kg, %)], lean mass [LM (kg, %)], and bone mineral content (g) was provided by enCORE v16 (GE Healthcare, Chicago, IL, United States). As FSHD is primarily a disease that affects the upper extremity, differences between upper and lower lean and fat mass were also obtained. Appendicular lean mass (ALM) was quantified as the sum of fat- and bone-free tissue in the arms and legs, and was normalized to height to control for fluctuations in body size (Merlini et al., 2014). An appendicular lean mass index [ALMI; ALM weight (kilograms (kg)]/height^2^ [meters (m), m^2^)] was utilized as an index of sarcopenia (Merlini et al., 2014), whereby the presence of sarcopenia was defined by an ALMI that is two standard-deviations lower than ALMI from the means observed in sex-specific reference groups (Baumgartner et al., 2004). Sarcopenic obesity was defined by the combined presentation of an ALMI of < 7.26 kg/m^2^ and proportion of whole body FM to whole body total mass (% FM) of > 28%, or an ALMI of < 5.45 kg/m^2^ and % FM of > 40%, in men and women, respectively (Baumgartner et al., 2004). Body mass index (BMI) was calculated from manual measurements of height (m) and weight (kg); study participants were categorized by BMI status into standard body composition categories (World Health Organization, 2019).

### Statistical Analysis

Data is reported as group averages (mean ± standard deviation), distribution normality was assessed and parametric vs. non-parametric methods were used as appropriate. Independent samples *t*-tests were used to compare differences in body composition between FSHD and control participants; in cases where the data was not normally distributed, the Mann-Whitney *U*-test was performed. Pearson product moment correlation was used to determine relationships between continuous variables. Statistical analyses were performed using SPSS v24.0 (SPSS, Inc., Chicago, IL, United States) with significance defined as an α-level of *p* < 0.05 for all comparisons.

## Results

### Subject Characteristics

FSHD and control participants were similar in age, weight, height, and BMI (*p* > 0.05 for all, Table 1). In the FSHD group, all 10 participants self-reported as non-Hispanic (NH) White; among controls, self-reported race was as follows: NH White: 7 (5 men, 2 women), Black: 1, Asian: 1, Hispanic: 1. Overall, the whole body total mass [calculated as sum of whole body fat mass (kg), whole body lean mass (kg), and bone mineral content (kg)] of individuals with FSHD was similar to that of healthy controls (84.5 ± 12.9 vs. 81.8 ± 13.5 kg, respectively, *p* = 0.65, Table 2).

**TABLE 1:** Subject characteristics.

	FSHD	Range (FSHD)	Control	Range (control)	*p*-value
**Age (years)**					
Males and females combined	50 ± 11	36–72	47 ± 13	31–75	0.60
Males	51 ± 12	36–72	48 ± 14	31–75	0.68
Females	45 ± 9	38–51	41 ± 14	31–51	0.80
**Weight (kg)**					
Males and females combined	85.4 ± 12.9	65.5–105.7	81.8 ± 13.4	65.3–103.7	0.55
Males	86.8 ± 12.0	67.9–105.7	81.6 ± 15.1	65.3–103.7	0.46
Females	79.8 ± 20.1	65.5–94.0	82.7 ± 4.5	79.5–85.9	0.87
**Height (m)**					
Males and females combined	1.80 ± 0.07	1.67–1.91	1.74 ± 0.08	1.63–1.78	0.09
Males	1.84 ± 0.04	1.78–1.91	1.76 ± 0.09	1.68–1.87	0.06
Females	1.70 ± 0.04	167–1.73	1.70 ± 0.10	1.63–1.78	0.95
**BMI (kg/m^2^)**					
Males and females combined	26.1 ± 4.4	21.4–33.7	26.7 ± 3.6	21.5–32.2	0.77
Males	25.7 ± 3.7	21.4–29.9	26.2 ± 3.4	21.5–31.8	0.80
Females	27.8 ± 8.3	21.9–33.7	28.7 ± 5.0	25.1–32.2	0.92

*Individuals with FSHD and controls were similar in age, height, weight and body mass index (BMI). kg: kilograms; m: meters; data are presented in mean ± SD.*

**TABLE 2:** Measures of body composition.

	FSHD	Range (FSHD)	Control	Range (control)	*p*-value
**Measures of sarcopenia**					
*ALM (kg)*					
Males and females combined	20.5 ± 4.4	15.3–27.5	26.5 ± 5.9	20.6–39.0	0.02
Males	21.2 ± 4.7	15.3–27.5	27.7 ± 6.1	20.6–39.0	0.03
Females	17.7 ± 1.9	16.4–19.1	22.0 ± 1.1	21.1–22.8	0.15
**Additional measures of lean mass**					
*Whole body lean mass (kg)*					
Males and females combined	47.6 ± 6.0	40.5–57.3	56.6 ± 11.1	45.9–78.8	0.05
Males	49.1 ± 5.8	40.5–55.0	58.8 ± 11.5	45.9–78.8	0.05
Females	41.7 ± 1.7	42.9–57.3	47.7 ± 0.6	47.2–48.1	0.10
*Total arms lean mass (kg)*					
Males and females combined	5.4 ± 1.1	43.9–73.9	7.5 ± 2.1	4.4–12.5	< 0.01
Males	5.7 ± 1.1	44.6–73.9	8.1 ± 2.1	5.1–12.5	0.01
Females	4.4 ± 0.04	43.9–44.5	5.5 ± 0.6	4.4–5.9	0.22
*Total legs lean mass (kg)*					
Males and females combined	15.1 ± 3.5	10.8–20.5	19.0 ± 3.9	14.0–26.5	0.03
Males	15.5 ± 3.8	10.8–20.5	19.6 ± 4.2	14.0–16.5	0.06
Females	13.3 ± 2.0	11.9–14.7	16.4 ± 0.6	16.0–16.8	0.25
**Additional measures of adiposity**					
*Whole body fat mass (kg)*					
Males and females combined	33.7 ± 10.1	21.8–49.6	22.0 ± 6.9	11.2–49.6	<0.01
Males	33.5 ± 9.4	21.8–49.6	19.7 ± 5.3	11.2–49.6	<0.01
Females	34.7 ± 17.0	22.7–46.7	31.1 ± 4.9	27.7–34.7	0.82
*Total arms fat mass (kg)*					
Males and females combined	3.2 ± 0.9	2.1–5.0	2.5 ± 0.9	1.4–4.9	0.09
Males	3.1 ± 0.9	2.1–5.0	2.2 ± 0.5	1.4–4.9	0.02
Females	3.4 ± 1.2	2.5–4.3	3.8 ± 0.6	3.4–4.2	0.75
*Total legs fat mass (kg)*					
Males and females combined	10.7 ± 2.4	7.2–14.9	6.0 ± 1.6	3.1–12.1	<0.01
Males	10.3 ± 2.1	7.2–13.1	5.4 ± 1.1	3.1–12.1	<0.01
Females	12.2 ± 3.9	9.4–14.9	8.5 ± 0.1	8.4–8.5	0.40

*When males and females are combined, individuals with FSHD had lower appendicular lean mass (ALM), arm lean mass and leg lean mass but greater body and leg fat mass. When separated by sex, only males with FSHD exhibited a significant difference in these measures. kg: kilogram; data are presented in mean ± SD.*

### Measures of Sarcopenic Obesity

**FIGURE 1:**
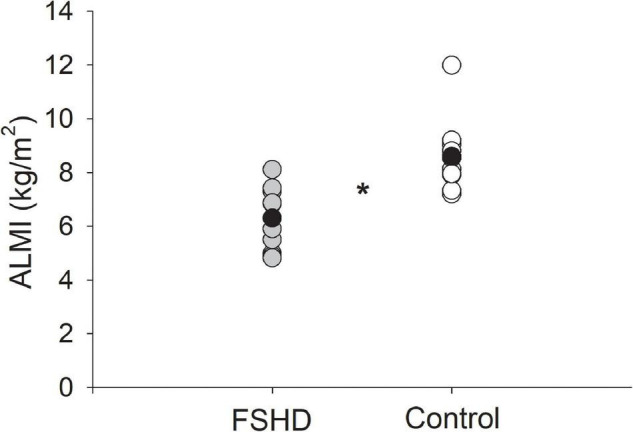
Appendicular lean mass index (ALMI, kg/m^2^) in FSHD and controls. Individuals with FSHD had lower lean mass as compared to controls. Black circles indicate average data for each group. *p < 0.01.

**FIGURE 2:**
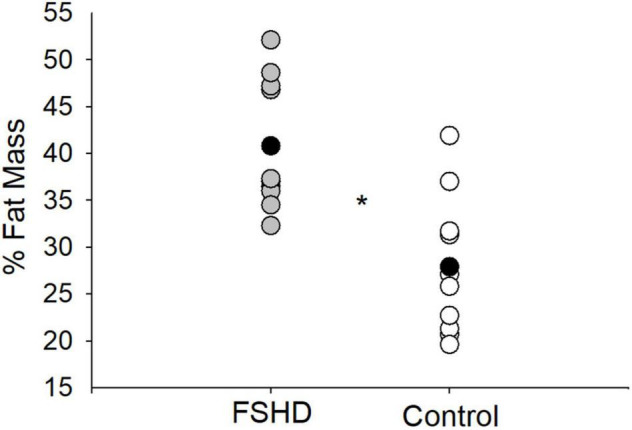
% fat mass in FSHD and controls. Individuals with FSHD had a higher % fat mass, as compared to controls. Black circles indicate average data for each group. *p < 0.01.

Adults with FSHD were found to have an ALM that was 23% lower, as compared to the control group (*p* = 0.02, Table 2). This observation was further accompanied by an ALMI score that was 27% lower among individuals with FSHD, as compared to healthy controls (*p* < 0.01, Figure 1). Furthermore, % FM was 46% greater in FSHD, compared with controls (*p* < 0.01, Figure 2). Mean alterations in ALMI (6.3 ± 1.3 kg/m^2^) and % FM (40.0 ± 6.4%) among men with FSHD were sufficient to meet the diagnostic criteria for sarcopenic obesity; furthermore, six of eight men with FSHD were individually found to meet compositional requirements for the condition. Conversely, the same criteria were not met in female FSHD counterparts (ALMI: 6.2 ± 1.0 kg/m^2^, % FM: 44.1 ± 11.4%), and neither of the two FSHD females individually met the diagnostic requirements. Sarcopenic obesity was not observed in any of the control participants.

### Additional Measures of Lean Mass

**FIGURE 3:**
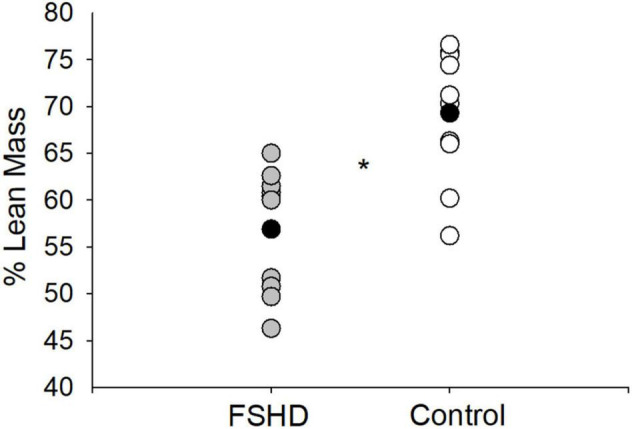
% lean mass in FSHD and controls. Individuals with FSHD had a lower % lean mass, as compared to controls. Black circles indicate average data for each group. *p < 0.01.

Additional measures of LM are located in Table 2. The absolute volume of whole body LM was 15% lower in FSHD, compared with controls, trending toward significance (*p* = 0.05). In addition, individuals with FSHD demonstrated a relative proportion of whole body LM to whole body total mass (% LM) that was 18% lower, compared with controls (*p* < 0.01, Figure 3). Furthermore, both the absolute volume of total arm (*p* < 0.01) and total leg *(p* = 0.03) LM were lower in FSHD by 29 and 21%, respectively, as compared with controls.

### Additional Measures of Adiposity

Additional measures of adiposity are located in Table 2. Absolute volume of whole body FM was 53% greater in FSHD compared with controls (*p* < 0.01). While the absolute volume of total leg FM among individuals with FSHD was 78% greater (*p* < 0.01), the arms were somewhat less affected, with mean total arm FM only 28% greater in the FSHD group, which did not reach statistical significance (*p* = 0.09).

### Self-Reported Measures of Functional Ability and Severity of Disease

Self-reported FSHD Health Index (HI) scores are located in Table 3, and range in value from 8.0 to 53.4 arbitrary units. Total FSHD-HI (*r* = −0.60), mobility and ambulation (*r* = −0.50), and activity limitation (*r* = −0.62) trended to be correlated with ALMI (*p* = 0.07), but not % FM or BMI (*p* > 0.05 for all). Activity limitation also trended to be correlated with age (*r* = 0.62, *p* = 0.06). Other self-reported measures included an attenuated amount of physical activity completed each day among individuals with FSHD (activity metabolic index score; FSHD: 28.0 ± 33.6 kcal/day, control: 184.3 ± 152.7 kcal/day; *p* < 0.01). Activity metabolic index score was not related to measures of body composition (% FM, ALMI) in either FSHD or control groups (*p* > 0.05 for all).

**TABLE 3:** FSHD Health Index (HI) Survey mean scores.

FSHD health index category	Mean score (*n* = 10)
Shoulder and arm function	53.4 ± 27.4
Mobility and ambulation	48.3 ± 22.3
Fatigue	44.9 ± 15.9
Social performance	44.0 ± 24.6
Core strength and function	43.0 ± 19.8
Activity limitation	40.1 ± 14.6
Body image	35.0 ± 22.1
Social satisfaction	33.1 ± 22.8
Emotional health	28.6 ± 18.5
Pain	19.9 ± 11.8
Hand and finger function	19.7 ± 22.3
Communication	19.5 ± 19.9
Gastrointestinal function	10.3 ± 14.8
Cognitive function	8.0 ± 12.8
Total FSHD-HI Score	20.1 ± 8.8

*Data are presented in mean ± SD.*

## Discussion

This study is the first to confirm the presence of sarcopenic obesity among individuals with FSHD, as reflected by a mean ALMI and % FM of < 7.26 kg/m^2^ and > 28% in afflicted males; furthermore, we are the first to show that individuals with FSHD exhibit the anatomic characteristics of sarcopenic obesity more often than age- and sex-matched controls. These observations are consistent with previous reports of significant alterations in body composition in people with FSHD, including widespread increases in measures of adiposity and reductions in lean mass (Skalsky et al., 2008; Orrell, 2011; Janssen et al., 2014).

### Sarcopenic Obesity in Muscular Dystrophy

Sarcopenic obesity is commonly found in other forms of muscular dystrophy, but has yet to be observed in FSHD. In fact, previous research has confirmed the presence of sarcopenic obesity among individuals with Bethlem myopathy, Ullrich congenital muscular dystrophy, rigid spine syndrome, limb girdle MD type 2d, Duchenne MD, and Becker MD (Miscione et al., 2013; Merlini et al., 2014; Rodríguez et al., 2017). Consistent with our findings, alterations in body composition – including greater FM and lower LM – have been reported among individuals with FSHD (Skalsky et al., 2008). In fact, Skalsky et al. (2008) noted a whole body LM value that was 17% lower in people with FSHD, as compared to control groups, a value which nearly mirrored our own observations (whole body lean mass 15% lower in FSHD group). Additionally, our findings of a % FM that was 53% greater among people with FSHD was similar to that reported by Skalsky et al. (2008), in which whole body % FM was found to be 41% higher in the clinical group. Interestingly, despite observations of a higher volume of FM, these authors noted a BMI that was similar between groups (Skalsky et al., 2008), a finding that was consistent with our study. Also in line with our research, Skalsky et al. (2008) reported significantly lower LM in the arms and legs. Similarly, observations of a greater volume of FM in the legs, but *not* in the arms, was also documented among people with FSHD. It is worthwhile to note that the DXA scanner utilized in the Skalsky et al. (2008) study compartmentalized the limbs into upper and lower portions (arm, forearm, thigh, leg), while the device used in our research provided more generalized values (arms, legs). Therefore, drawing exact comparisons between appendicular measures of LM and FM between the two studies is not possible. Finally, as Skalsky et al. (2008) did not assess ALM or ALMI in their study, we are not able to determine whether FSHD study participants met the diagnostic criteria for sarcopenic obesity, though as mean values of % FM in the FSHD group did not meet minimal threshold values (mean: 25.41 ± 8.33%) (Skalsky et al., 2008), the presence of the condition within this group of FSHD participants appears unlikely.

While the presence of sarcopenic obesity was only observed among male FSHD participants in our research, other studies have documented the condition among both sexes, in alternative forms of muscular dystrophy. In fact, in work by Miscione et al. (2013), in which 8 participants (male: 3, female: 5) with either Bethlem myopathy or Ullrich congenital muscular dystrophy were studied, sarcopenic obesity was confirmed in two of three males and all five females. Although the sample size was low in Miscione et al. (2013), they had three additional females, with all five meeting the criteria for sarcopenic obesity. Neither of our two females met the criteria which could indicate that the presence of sarcopenic obesity in different forms of muscular dystrophy is not equivocal. Notably, in adults without muscular dystrophy, it has been shown that females may be less, more, or as likely to develop sarcopenic obesity, as their male counterparts (Du et al., 2018). Among whites, which formed the majority of our study (85%), rates of sarcopenic obesity are similar between age-matched males and females (Du et al., 2018). However, sex-specific differences in FSHD phenotype presentation, in which females appear to be less affected than their male counterparts, have been reported (Zatz et al., 1998; Tonini et al., 2004). In our study, an elevated mean % FM of 44.1 ± 1.0% in the two females with FSHD did result in the fulfillment of one of the two objective requirements to meet the criteria for sarcopenic obesity (% FM: > 40%). However, we also observed a mean ALMI of 6.2 ± 1.0 kg/m^2^ in the females with FSHD, a value that exceeds the minimum threshold associated with presentation of the condition (ALMI: < 5.45 kg/m^2^). Therefore, although neither of the two females reached the diagnostic criteria for sarcopenic obesity, the small sample size in this study precludes any speculation regarding sex differences in sarcopenic obesity in adults with FSHD.

### Clinical Relevance of Sarcopenic Obesity

Identifying the presence of sarcopenic obesity in adults with FSHD is of high significance, as it may indicate an increased propensity toward greater impairments in functional capacity, and greater risk of morbidity and mortality. According to the Concord Health and Aging Project, sarcopenic obesity is linked to an increased risk of frailty and instrumental activity of daily living disability (IADL), a measure that is characterized by an inability to perform tasks for independent living (Hirani et al., 2017). Furthermore, research by Baumgartner note that older men (>60 years of age) with sarcopenic obesity are eight times more likely to develop three or more disabilities than age- and sex-matched controls (Baumgartner, 2000). This observation is even more striking among older females with sarcopenic obesity, in which the risk for multiple disabilities was increased by a factor of 11 (Baumgartner, 2000). In addition, the relationship between impaired physical function and sarcopenic obesity appears to be stronger than an association with either obesity or sarcopenia alone (Baumgartner, 2000), thereby highlighting the cumulative effect of these factors on functionality in an aging population. Overall, these reports are in line with our own findings, in which a trending relationship between markers of sarcopenic obesity and self-reported impairments in mobility and ambulation or activity limitation was observed. While it is difficult to know whether sarcopenic obesity is more influential than FSHD when it comes to the etiology of physical impairment, these observations suggest that, at a minimum, it likely compounds the physical disability that individuals with FSHD already experience (Kilmer et al., 1995).

While DXA scanning is widely believed to be the gold-standard in body composition assessment, BMI charts are frequently used to estimate % FM in community settings and among older adults (Kuczmarski et al., 1994; Baumgartner et al., 1995). However, the relationship between % FM and BMI is believed to be significantly influenced by age and sex, and may not be the best indicator of total body fatness (Gallagher et al., 1996). In our research, BMI was similar between FSHD and controls, but fat mass was significantly greater in people with FSHD. Furthermore, despite trending relationships between measures of sarcopenic obesity and physical function, an association between BMI and these same parameters was absent. Together, these findings cast doubt on the relevancy of using BMI as a valid tool for physical and functional assessment among people with FSHD. In final, DXA is a cost-effective and time efficient imaging modality that can be utilized as a measurement of disease severity and disease progression for future clinical trials.

### Mechanisms Contributing to Sarcopenic Obesity

Though complex in nature, sarcopenic obesity is believed to be driven by a synergistic combination of biologic, hormonal, and behavioral influences. In fact, the atrophy and loss of type II muscle fibers – a phenomenon that is believed to be predominantly responsible for the presentation of sarcopenia (Walrand et al., 2011) – is reportedly caused by an amalgamation of factors, including neurodegenerative processes within spinal α-motor neurons, dysregulation of anabolic hormone production (insulin, growth, and sex hormones), and inadequate nutritional intake (Walrand et al., 2011). Furthermore, sarcopenia is believed to be mediated by a deconditioned state (Walrand et al., 2011), and the presence of a significantly reduced physical activity score in the FSHD cohort in our study suggests that deconditioning may have been a precipitating factor in the manifestation of sarcopenia. It is worthwhile to note that while individuals with FSHD have been reported to exhibit reductions in both type IIa and IIx muscle fibers (Celegato et al., 2006), it is unclear whether this phenomenon is caused by the factors described above, or is purely an intrinsic result of the disease itself. As such, individuals with FSHD exhibit indirect evidence of DUX4 protein expression in muscle biopsies (Yao et al., 2014), which leads to an inability to properly replace diseased or damaged muscle tissue with new myofibers (Bosnakovski et al., 2018) which may lead to a pro-adipogenic state within muscle of adults with FSHD. In addition, physical inactivity has been widely cited as a contributor in the development of obesity (Myers et al., 2017). Observations of physical inactivity have been noted in other forms of muscular dystrophy, whereby 44% of people with limb-girdle or Charcot-Marie-Tooth muscular dystrophy exhibit an inability to meet minimum threshold recommendations for daily exercise (Andries et al., 2020). Thus, the physical *inactivity* demonstrated in our FSHD cohort likely not only contributed to sarcopenia, but also obesity. The lack of a correlation between either physical activity and % FM (*p* = 0.79) or physical activity and ALMI (*p* = 0.15), among people with FSHD, however, suggests that the inherent influence of FSHD likely plays a significant role in the etiology of sarcopenic obesity.

### Limitations

Limitations of this study should be considered when interpreting the data. The small sample size in female participants, likely contributed to an inability for the females on average to meet the diagnostic criteria for sarcopenic obesity. Previous research by Miscione et al. (2013) has confirmed the presence of sarcopenic obesity in both male and female dystrophic groups, but not specifically in FSHD. Furthermore, as we did not control for clinical severity within the FSHD group, it is possible that our female FSHD participants exhibited a lesser-degree of disease than their male counterparts, whereby the presence of anatomic alterations in body composition were not yet manifest, a theory which has been supported by previous research (Zatz et al., 1998; Tonini et al., 2004). Since our study used a DXA scan and not MRI imaging to assess body composition, we were unable to assess whether differences in % FM between FSHD and control groups were driven by general increases in adiposity, or by intramuscular fat infiltration, a finding which has been previously reported (Janssen et al., 2014), and which is believed to be a hallmark characteristic of FSHD. Finally, while all FSHD study members were Caucasian, 3 of 10 control participants (all men) were of differing races, a factor that may have a confounding effect on study outcomes. Though alterations in body composition between races have been widely reported (Heymsfield et al., 2016), it appears that these differences may be driven by sex, whereby variances are noted primarily among female racial groups (Grumstrup-Scott and Marriott, 1992). According to research by Gerace et al. (1994), measures of total body fat and fat-free mass are similar between Black and White males, though whether the same is true between White and Hispanic or Asian males remains to be elucidated. Finally, because of the case-control study design, we are unable to establish a causative relationship between FSHD and presence of sarcopenic obesity.

## Conclusion

This research is the first to show that men with FSHD meet the diagnostic criteria for sarcopenic obesity more often than age-matched controls. Sarcopenic obesity is a complex medical condition, which manifests as a number of both acute and long-term implications. Identifying individuals that may be at an increased risk for sarcopenic obesity, will lead to preventative rehabilitative strategies to reduce the prevalence of the condition, among individuals of all ages and health statuses. Furthermore, by identifying sarcopenic obesity as a comorbidity of FSHD, we have taken a major step forward in understanding the anatomic and physiologic contributions to impaired health and physical function in this genetic disease. Future research in this area should focus on strategies (i.e., exercise) to address the sarcopenic obesity-driven losses in functionality, and improve quality of life among individuals with neuromuscular impairment.

## Data Availability Statement

The raw data supporting the conclusions of this article will be made available by the authors, without undue reservation, to any qualified researcher.

## Ethics Statement

The studies involving human participants were reviewed and approved by the University of Minnesota Institutional Review Board. The patients/participants provided their written informed consent to participate in this study.

## Author Contributions

KV and MM completed the data collection and organization. KV was also responsible for statistical analysis and manuscript conceptualization and writing. MK and MK-R were responsible for conceptual design of study and were primary editors of the publication. MK-R also served in an advisory capacity. All authors contributed to the article and approved the submitted version.

## Conflict of Interest

The authors declare that the research was conducted in the absence of any commercial or financial relationships that could be construed as a potential conflict of interest.
