# Corrigendum: Sarcopenic Obesity in Facioscapulohumeral Muscular Dystrophy

**DOI:** 10.3389/fphys.2022.908605

**Published:** 2022-04-28

**Authors:** Kathryn Vera, Mary McConville, Michael Kyba, Manda Keller-Ross

**Affiliations:** ^1^ Division of Rehabilitation Science, University of Minnesota, Minneapolis, MN, United States; ^2^ Health and Human Performance Department, University of Wisconsin—River Falls, River Falls, WI, United States; ^3^ College of Saint Benedict, St. Joseph, MN, United States; ^4^ Lillehei Heart Institute and Department of Pediatrics, University of Minnesota, Minneapolis, MN, United States; ^5^ Division of Physical Therapy, University of Minnesota, Minneapolis, MN, United States

**Keywords:** FSHD, muscular dystrophy, sarcopenic obesity, appendicular lean mass index, body composition

In the original article, there was a mistake in Figure 2 and Figure 3 as published. The Y axes were switched for the figures. The corrected Figures 2, 3 appear below.

**FIGURE 2:**
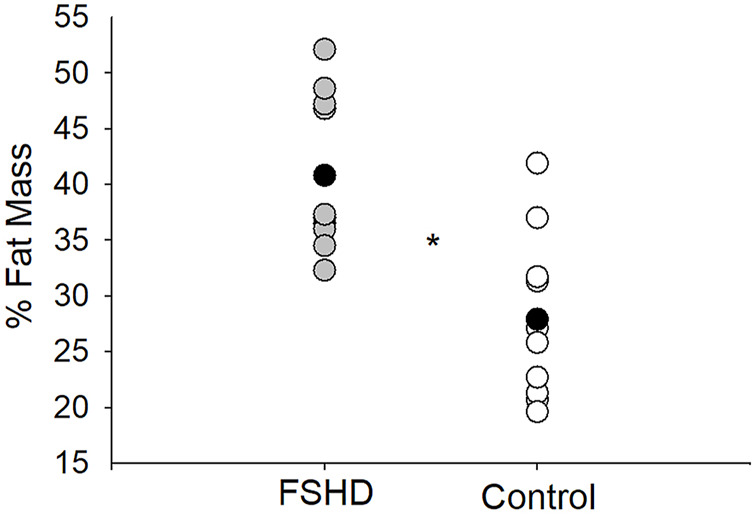
% fat mass in FSHD and controls. Individuals with FSHD had a higher % fat mass, as compared to controls. Black circles indicate average data for each group. *p < 0.01.

**FIGURE 3:**
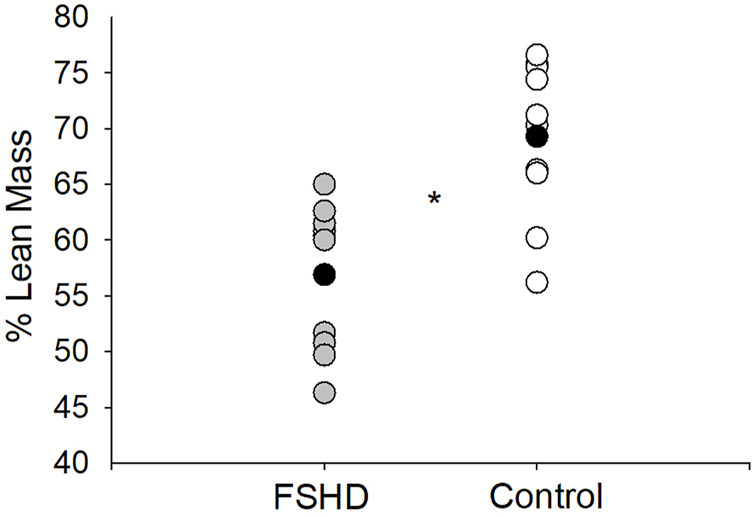
% lean mass in FSHD and controls. Individuals with FSHD had a lower % lean mass, as compared to controls. Black circles indicate average data for each group. *p < 0.01.

The authors apologize for this error and state that this does not change the scientific conclusions of the article in any way. The original article has been updated.

